# The Role of IL-23 Inhibitors in Crohn’s Disease

**DOI:** 10.3390/jcm13010224

**Published:** 2023-12-30

**Authors:** Jacopo Fanizza, Ferdinando D’Amico, Francesca Lusetti, Ernesto Fasulo, Mariangela Allocca, Federica Furfaro, Alessandra Zilli, Tommaso Lorenzo Parigi, Simona Radice, Laurent Peyrin-Biroulet, Silvio Danese, Gionata Fiorino

**Affiliations:** 1 Department of Gastroenterology and Endoscopy, IRCCS San Raffaele Hospital, Vita-Salute San Raffaele University, 20132 Milan, Italy; fanizza.jacopo@hsr.it (J.F.); damico.ferdinando@hsr.it (F.D.); francesca.lusetti@gmail.com (F.L.); fasulo.ernesto@hsr.it (E.F.); allocca.mariangela@hsr.it (M.A.); furfaro.federica@hsr.it (F.F.); zilli.alessandra@hsr.it (A.Z.); parigi.tommaso@hsr.it (T.L.P.); radice.simona@hsr.it (S.R.); danese.silvio@hsr.it (S.D.); 2 Department of Biomedical Sciences, Humanitas University, 20089 Milan, Italy; 3 Department of Gastroenterology, IRCCS Policlinico San Matteo, University of Pavia, 27100 Pavia, Italy; 4 Department of Gastroenterology, Nancy University Hospital, F-54500 Vandœuvre-lès-Nancy, France; peyrinbiroulet@gmail.com; 5 Inserm, NGERE, University of Lorraine, F-54000 Nancy, France; 6 INFINY Institute, Nancy University Hospital, F-54500 Vandœuvre-lès-Nancy, France; 7 FHU-CURE, Nancy University Hospital, F-54500 Vandœuvre-lès-Nancy, France; 8 Groupe Hospitalier Privé Ambroise Paré-Hartmann, Paris IBD Center, F-92200 Neuilly-sur-Seine, France; 9 Division of Gastroenterology and Hepatology, McGill University Health Centre, Montreal, QC H4A 3J1, Canada; 10 IBD Unit, Department of Gastroenterology and Digestive Endoscopy, San Camillo-Forlanini Hospital, 00152 Rome, Italy

**Keywords:** inflammatory bowel disease, Crohn’s disease, IL-23 inhibitors, risankizumab, guselkumab, mirikizumab, brazikumab

## Abstract

Promoting a Th17 pathogenic response, the interleukin (IL)-23 pathway is crucial in the pathophysiology of inflammatory bowel disease (IBD). With a favorable safety profile, ustekinumab, a monoclonal antibody targeting the shared p40 component of IL-12/23, is currently approved for the treatment of IBD in patients with disease refractory to corticosteroids and biologic drugs. Risankizumab, mirikizumab, and guselkumab are specific IL-23p19 antagonists tested for the treatment of Crohn’s disease (CD). However, only risankizumab currently has been approved for its treatment. Trials with guselkumab and mirikizumab are currently ongoing, with promising preliminary efficacy and safety results. In this review, we provide a summary of the current knowledge about selective IL-23 inhibitors, focusing on their positioning in the therapeutic algorithm of patients with moderate to severe CD.

## 1. Introduction

Inflammatory bowel diseases (IBDs), namely Crohn’s disease (CD) and ulcerative colitis (UC), are chronic and disabling immune-mediated conditions affecting the gastrointestinal tract, with a remitting and relapsing course [1]. The pathogenesis is multifactorial, probably triggered by a dysregulated immune response to gut microbiota in genetically susceptible individuals [2,3]. Traditionally, the management of IBD patients included a broad spectrum of anti-inflammatory drugs, such as 5-aminosalicylic acid drugs, steroids, and non-targeted immunosuppressants [4,5]. In the last two decades, given an increased understanding of the pathogenic mechanisms underlying these diseases, there has been a revolution in therapy, which to date includes biological therapies and small molecules targeting the adaptive immune system [6]. Since the late 1990s, the advent of TNF inhibitors (infliximab and adalimumab) led to a breakthrough in treatment and disease control: infliximab demonstrated excellent efficacy in inducing and maintaining remission, preventing disease complications, and achieving mucosal healing [7]. On the other hand, literature data show that up to 30% of patients do not respond to initial therapy with TNF inhibitors and that up to 50% lose response over time, with 10% of patients still requiring surgery [8]. Thus, there is an urgent need to develop new molecules directed against different molecular targets. New drugs with different signaling pathways for intestinal inflammation have been developed, targeting for instance α4β7 integrin, Janus kinase (JAK), and interleukin 12 and 23 (IL-12/IL-23). In particular, studies on mice models showed that IL-12 played a crucial role in several inflammatory conditions, including colitis [9]: thus, new molecules directed against this target were developed. IL-12 and IL-23 are involved in promoting inflammatory responses. IL-12 is a heterodimer composed of a p35 and p40 subunit, and it is primarily produced by dendritic cells, macrophages, and neutrophils in response to antigens. IL-12 plays a pivotal role in guiding T-cell differentiation toward the Th1 interferon-gamma-producing lineage. IL-23 consists of a complex formed by an IL12p40 subunit paired with a distinct IL-23p19 unit through a disulfide bond [10,11]. IL-23 has a major role in the differentiation of Th-17 cells; in particular, when it interacts with its receptor, it activates the inflammatory cascade through a pathway mediated by the Janus kinase (JAK) and the signal transducer and activator of transcription (STAT) [12]. IL-23 plays a crucial role in the regulation of the gut immune response, and its dysregulation may contribute to the development or exacerbation of inflammatory conditions [10,13].

Such evidence was a turning point in our knowledge of the pathogenesis of IBD and led to a growing interest in selectively blocking IL-23 [12,14]. In recent years, efforts have been directed toward the development of increasingly selective drugs in order to minimize the risk of off-target/side effects and maximize efficacy [15].

First-generation treatment entails a therapy directed at anti-IL-12p40, with its effectiveness attributed to the inhibition of IL-23 rather than the direct blocking of IL-12 [11]. The only real-world experience in such therapies comes mainly from ustekinumab (a p40 subunit IgG1k inhibitor of IL12/23), which was initially approved in 2009 for psoriasis and, with regard to IBD, since 2016 for CD and since 2019 for UC, demonstrating an excellent efficacy and safety profile for both diseases [16,17]. On the other hand, it is uncertain whether its effects are primarily attributed to the inhibition of IL-12, IL-23, or both [18].

The second generation of selective anti-IL-23 therapies targets IL-23p19, and they have demonstrated greater effectiveness compared to ustekinumab in treating other immune-mediated conditions, such as plaque psoriasis, prompting us to explore their potential use in IBD [19,20].

In addition, the rationale for specifically targeting the p19 subunit of IL-23 is to enhance safety by preserving the normal IL-12-mediated Th1 immune response, which is crucial for defending against intracellular pathogens like those that trigger interferon-gamma release from T and NK cells [21]. This approach aims to maintain the efficacy achieved with p40 antibodies while avoiding potential disruptions to the immune response [18]. However, although they belong to the same category of drugs, they have different molecular attributes that may lead to differences in clinical efficacy. In vitro studies have shown that guselkumab, for example, has the ability to bind CD64+ myeloid cells, which are elevated in the inflamed colon during IBD and are associated with the severity of endoscopic disease [22]. This binding occurs through its Fc region, and guselkumab exhibits a higher affinity and potency in neutralizing IL-23 compared to risankizumab [23].

The IL-12-mediated Th1 response has been suggested to have a more significant impact on susceptibility to certain diseases, such as mycobacterial infections, compared to the IL-23-mediated Th17 response [24]. Similar relationships have been observed with other pathogens, including *Pneumocystis jiroveci*, *Cryptococcus neoformans*, and *Toxoplasmosis gondii*. By selectively targeting IL-23p19 while leaving IL-12 intact, host immunity against a variety of pathogens could be preserved [11,18].

The aim of this review is to summarize the available data on ‘second generation’ selective IL-23 inhibitors in patients with CD in order to better understand how to position these drugs into the therapeutic algorithm of CD.

## 2. Materials and Methods

We searched the Pubmed, Embase, and Scopus databases up to 30 October 2023 in order to identify studies reporting the efficacy and safety data of selective IL-23 inhibitors in patients with CD. The following search terms were used: ‘IL-23 inhibitors’, ‘brazikumab’, ‘risankizumab’ ‘guselkumab’, and ‘mirikizumab’ combined with ‘Crohn’s disease’ and ‘inflammatory bowel disease’. Only articles published in English were considered. Three authors (FJ, LF, and DF) independently reviewed titles and abstracts to identify eligible studies. The full texts of the selected articles were examined for inclusion, and relevant references in their lists were hand-searched to identify studies missed by the electronic search. Abstracts and articles were included based on their relevance.

## 3. Pharmacodynamic

The interleukin 12 (IL-12) family consists of four members (IL-12, IL-23, IL-27, and IL-35). Each cytokine is heterodimeric and consists of an α chain (p19, p28, or p35) and a β chain (p40 or Ebi3) [25].The four-helix bundle structure of alpha chains are a hallmark of the IL-6 superfamily, to which the IL-12 family belongs. The ß chains, on the other hand, have homology with soluble class I cytokine receptor chains like IL-6Rα. While Ebi3 can pair with either p28 or p35 to produce IL-27 or IL-35, respectively, the p40 chain can pair with either p35 or p19 to form IL-12 or IL-23 [26].

Antigen-presenting cells (APCs), such as dendritic cells and macrophages, generate IL-12 and IL-23 in response to early innate signals [27]. While IL-12 appears to act mainly on naïve T lymphocytes [28], IL-23 was first thought to affect memory T cells only and not naive T cells. Later, it was discovered that IL-23 encouraged T helper 17 cell proliferation and maintenance but not differentiation [27]. Through the production of IL-17A, IL-17F, IL-22, IL-26, TNF, and IFNγ, these cells are implicated in immune responses to bacteria and fungi and have been identified as important autoimmune mediators [29].

IL-12 signals via IL12Rβ1 and IL12Rβ2 receptors [30], whereas IL-23 uses IL12Rβ1 and IL23R receptors for signaling [31]. Members of the Janus kinase–signal transducers and activators of transcription (JAK-STAT) family mediate signaling through each of these receptors [32]. When IL-12 binds its receptor, it induces activation of the kinases JAK2 and TYK2, with subsequent phosphorylation of the transcription factor STAT4, which initiates the IL-12 gene program [33]. Instead, when IL-23 binds its receptor, STAT3 is phosphorylated, and this leads to the different results from signaling downstream of these cytokines [34].

**Figure 1:**
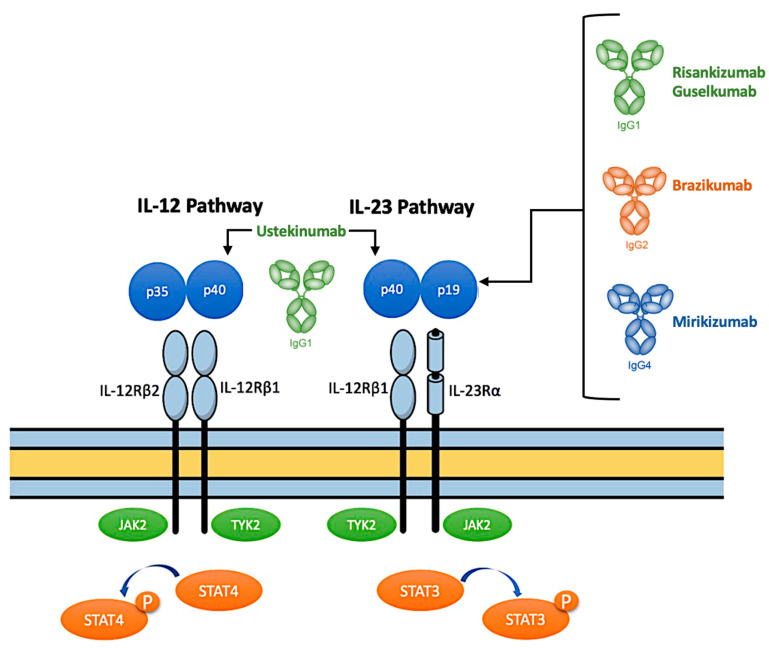
The interleukin (IL)-12/IL-23 pathway.

IL-23, more than IL-12, has a pivotal role in the pathogenesis of IBD [27]. Of note, IL-12 expression is increased in lamina propria mononuclear cells from CD patients. Moreover, IL-12 may further stimulate lamina propria T-cell inflammatory responses [35]. Inflammatory effects of IL-23 can be mediated by pathogenic TH17 cells, innate lymphoid cells, and macrophages [36], whereas the protective benefits of IL-23 can be mediated by epithelial cells, antimicrobial pathways, and downregulation of TH1 cells [37]. For these reasons, the selective antagonism of this cytokine may have a crucial role in the therapy of CD (Figure 1).

## 4. Results

### 4.1. Risankizumab

#### 4.1.1. Efficacy

Risankizumab is a humanized IgG1 monoclonal antibody already approved in 2019 for the treatment of moderate-to-severe plaque psoriasis [38].

It was initially studied for the treatment of moderate-to-severe CD in a phase II, proof of concept, multicenter, randomized, double-blind, placebo-controlled induction trial [39].

In this trial, 121 patients were enrolled; 93% of them had failed to respond to previous treatment with TNF-antagonists or vedolizumab and with moderate-to-severe CD defined by a Crohn’s disease endoscopic index of severity (CDEIS) ≥7 or ≥4 for isolated ileitis and a CDAI score between 220 and 450. These patients were randomized 1:1:1 to receive intravenous (IV) risankizumab (600 mg), risankizumab (200 mg), or a placebo once daily at week 0, 4, and 8 for an induction period of 12 weeks. The primary endpoint of clinical remission, defined by a CDAI < 150 at week 12, was achieved in 24% (200 mg group) and 37% (600 mg group) of patients treated with the drug compared with 15.4% in the placebo arm (*p* = 0.0489). Clinical response (CDAI < 150 or decrease of ≥100 points), endoscopic remission (CDEIS ≤ 4 or ≤2 for isolated ileitis), endoscopic response (decrease ≥50% of CDEIS), and deep remission (clinical plus endoscopic remission) were the secondary endpoints of the study. A higher rate of patients on risankizumab therapy achieved secondary endpoints: for clinical response: 31% in the pooled risankizumab dose groups vs. 15% of the placebo group (*p* = 0.0489); for endoscopic remission, 17% vs. 3% (*p* = 0.0015); for endoscopic response, 32% vs. 13% (*p* = 0.0104); while for deep remission, 7% vs. 0% of the placebo arm (*p* = 0.0107) [39].

In an open-label extension of the study, patients who did not achieve clinical remission and endoscopic remission at week 12 received open-label IV therapy with risankizumab (600 mg) every 4 weeks for 12 weeks, while those who did reach deep remission entered a 12-week washout phase. At week 26, 55% of patients in the placebo group, 59% in the 200 mg group, and 47% in the 600 mg group achieved clinical remission [40].

At week 26, 62 patients who were in clinical remission started maintenance therapy with a 180 mg SC dose of risankizumab. At week 52, 71% of the patients were in clinical remission, 35% achieved endoscopic response, and 29% were in deep remission [40].

Finally, in an open-label extension study, 65 patients (61 patients from maintenance study in therapy with risankizumab (180 mg SC q8w) and 4 patients who had lost response in the parent study and were first reinduced with risankizumab (600 mg) every 4 weeks) were enrolled to receive the same subcutaneous (SC) therapy for a median of 33 months. The proportions of patients with clinical and endoscopic remission remained stable at the end of the observation period of 112 weeks (>71% and >42%, respectively) [41].

In 2022, further findings on the effectiveness and safety of risankizumab in CD were disclosed from two phase III trials.

The ADVANCE trial was a double-blind, randomized, placebo-controlled trial. The aim of the study was to evaluate efficacy as induction therapy for moderate or severe CD in patients who had shown intolerance or ineffectiveness to one or more approved biologics or conventional therapy [42]. A total of 850 patients were enrolled and randomized 2:2:1 to receive a placebo or 1200 mg or 600 mg of risankizumab IV at week 0, 4 and 8 (Table 1). The coprimary endpoints were clinical remission (defined as a CDAI less than 150 in the USA and an average daily liquid or very soft stool frequency of 2.8 or less plus an average daily abdominal pain score of 1 or less, with both not worse than the baseline in non-USA countries) and endoscopic response (defined as a greater than 50% decrease in SES-CD from the baseline or (for isolated ileal disease and a baseline SES-CD of 4) at least a 2-point reduction from the baseline) at week 12. At week 12, at all primary endpoints, risankizumab was superior to the placebo. In particular, CDAI clinical remission was achieved in 45% of patients in the risankizumab 600 mg arm (*p* < 0.0001) and in 42% of patients in the 1200 mg arm (*p* < 0.0001) versus the placebo (25%). Similarly, clinical remission based on patient-reported stool frequency and abdominal pain was achieved in 43% of patients in the risankizumab 600 mg arm (*p* < 0.0001) and in 41% of patients in the 1200 mg arm (*p* < 0.0001) versus the placebo (22%). A considerably higher percentage of patients receiving 600 mg (40%; *p* < 0.0001) or 1200 mg (32%; *p* < 0.0001) of risankizumab also experienced an endoscopic response at week 12 compared to the placebo (12%). When examining the same endpoints within specific patient subgroups (those with prior biologic treatment and those without), similar patterns of higher response rates compared to the placebo were observed. Specifically, for CDAI-based clinical remission, the rates were 49% and 47% for patients without previous biologic treatment as opposed to 43% and 38% for patients with prior biologic treatment for the 600 mg and 1200 mg arms, respectively. Likewise, for clinical remission based on stool frequency and abdominal pain scores, there were rates of 48% (600 mg group) and 44% (1200 mg group) for patients without previous biologic treatment and 41% (600 mg arm) and 39% (1200 mg arm) for patients with prior biologic treatment. Finally, in patients without prior biologic treatment failure, the endoscopic response rates were 50% and 44% for the risankizumab 600 mg and 1200 mg groups, respectively. Conversely, in patients with a history of previous biologic treatment failure, the rates were 33% (600 mg group) and 24% (1200 mg group) [42].

In the MOTIVATE induction trial, which was a multicenter, double-masked, randomized study, the primary endpoints were clinical and endoscopic remission, but only patients with a history of prior biological treatment failure were included in the enrollment. A total of 569 patients were randomized 1:1:1 to receive 600 mg or 1200 mg or a placebo every 4 weeks. The rates of CDAI clinical remission (42% in the 600 mg group (*p* < 0.0001) and 40% in 1200 mg group (*p* < 0.0001)) and stool frequency and abdominal pain score clinical remission were significantly higher in risankizumab group (35% in the 600 mg arm (*p* = 0.0007) and 40% in 1200 mg arm (*p* < 0.0001)) compared to the placebo (20% and 19%, respectively). Similar results were obtained in achieving the endoscopic response at week 12 (29% with *p* < 0.0001 and 34% with *p* < 0.0001 versus 11% of the placebo group). No significant difference in efficacy was observed between two groups for any coprimary endpoints in the ADVANCE or MOTIVATE trials [43].

Additionally, a considerably larger proportion of treated patients evidenced mucosal healing (defined as an ulcerated surface subscore of 0 in subjects with a subscore of ≥1 at the baseline) and endoscopic remission (defined as SES-CD ≤ 4 and at least a 2-point reduction versus the baseline and no subscore greater than 1 in any individual variable) during the ADVANCE and MOTIVATE induction investigations. Patients who received risankizumab (600 mg IV) experienced mucosal healing at week 12 at 21% (ADVANCE) and 14% (MOTIVATE), respectively, as opposed to 8% and 4% of patients in the placebo arm. At week 12, achieving endoscopic remission was observed in 24% of patients in the ADVANCE trial and 19% in the MOTIVATE trial for those receiving the drug. This stands in contrast to 9% and 4% in the respective placebo groups [44].

In the phase III FORTIFY trial, a multicenter, randomized, double-blind, placebo-controlled, maintenance withdrawal study, patients who had achieved clinical remission in the previous two trials were enrolled and randomized 1:1:1 into three arms: subcutaneous (SC) risankizumab (180 mg), SC risankizumab (360 mg), or a placebo every 8 weeks. The primary endpoints were clinical remission and endoscopic response at week 52. At week 52, 55% (*p* = 0.0031) and 52% (*p* = 0.0054) of patients in 180 mg and 360 mg groups achieved CDAI clinical remission compared to the placebo (41%). In contrast, the endoscopic response rates were 47% in the two arms of patients receiving treatment with risankizumab (*p* < 0.0001) versus 22% in the placebo arm [45]. Mucosal healing and endoscopic remission were observed during the FORTIFY maintenance study in patients treated with risankizumab (360 mg SC). Mucosal healing was observed at week 52 in 31% in the drug-treated arm compared with 10% in the placebo-treated arm (nominal *p* < 0.001), while endoscopic remission was achieved in 39% of patients treated with the same drug dose compared with 13% of patients receiving the placebo (nominal *p* < 0.001) [44].

Given its valuable results, risankizumab was approved in 2022 by both the FDA and EMA for the treatment of moderate-to-severe CD [44,46].

The role of risankizumab in the treatment of CD was also compared with ustekinumab. The SEQUENCE study (NCT04524611), a phase III clinical trial, was designed as a direct comparison between risankizumab and ustekinumab in the treatment of CD in adults who have previously not responded to one or more TNF inhibitors [47]. The outcomes for the primary endpoint, which assessed clinical remission, defined as a Crohn’s Disease Activity Index (CDAI) < 150 at week 24, demonstrated that risankizumab was non-inferior to ustekinumab, with a non-inferiority margin of 10%. Specifically, the remission rates were 59% in the risankizumab group and 40% in the ustekinumab group [48]. Regarding the second primary endpoint, which assessed endoscopic remission, defined as a Simple Endoscopic Score for Crohn’s Disease (SES-CD) ≤4 with at least a 2-point reduction compared to the baseline and no subscore greater than 1 in any individual component) at week 48, the results indicated that risankizumab was superior to ustekinumab. The remission rates were 32% in the risankizumab group and 16% in the ustekinumab group, with a statistically significant difference (*p* < 0.0001) [48].

#### 4.1.2. Safety

In the initial induction trial involving risankizumab for CD, the incidence of any AE was comparable across the three groups, with rates of 82% in the placebo group, 78% in the 200 mg group, and 76% in the 600 mg group [39].

In the open-label extension study, arthralgia (22% of patients), headache (20%), abdominal pain (18%), and nausea (16%) were the most common AEs, while severe adverse events (SAEs) (worsening of CD and intestinal obstruction) were observed in 11% of patients; serious infections were detected in 4% of patients [40].

The overall incidence of treatment-emergent AEs during and following the 12-week induction period in ADVANCE and MOTIVATE was comparable across all treatment groups. In the ADVANCE trial, the rates of incidence of AEs were 56%, 51%, and 56% for the 600 mg, 1200 mg, and placebo groups, respectively; and 48%, 59%, and 66% for the same group in the MOTIVATE trial. SAEs were detected in 7% and 4% vs. 15% and 5% and 4% vs. 13% in the ADVANCE and MOTIVATE trials. Only 1% of patients in every treatment group showed serious infections vs. 4% (ADVANCE trial) and 2% (MOTIVATE trial) of the placebo group. No cases of non-melanoma skin cancer, major adverse cardiovascular events (MACE), or death were recorded [42].

In the FORTIFY trial, the rates of AEs (72% in both risankizumab SC groups vs. 73% in the placebo SC group) and SAEs (12%, 13%, and 13%) were similar in all arms. The incidence of infectious adverse events was lower in both risankizumab treatment groups (34% for the risankizumab 180 mg and 360 mg groups) than in the withdrawal SC placebo group (40%). Serious infections were detected in 3%, 4%, and 4%, respectively, in the 180 mg, 360 mg, and placebo groups, with two and one cases of herpes zoster infection in the 180 mg and placebo arms. Only one malignancy (breast cancer) was observed in one patient in the 360 mg group, and it was considered by the investigator to be unrelated to the study drug. Finally, a MACE was reported during the maintenance study in the same group (a patient with a previous history of dyslipidemia and smoking) [45].

### 4.2. Brazikumab

#### 4.2.1. Efficacy

Brazikumab (MEDI2070) is a human IgG2 monoclonal antibody that binds exclusively the p19 subunit of IL-23, inhibiting the binding of IL-23 to its receptor. It has been tested in the treatment of CD in patients that had a primary and secondary non-response to TNF antagonists in a phase II trial (NCT01714726) [49]. In this trial, 119 patients were enrolled and randomized 1:1 to receive 700 mg IV of brazikumab at the baseline and at week 4 or a placebo. A CDAI decrease of 100 points from the baseline (clinical response) or a CDAI < 150 (clinical remission) at week 8 were the primary outcomes. At week 8, clinical response was observed in 49.2% of the treated patients vs. 26.7% of the patients receiving a placebo (*p* = 0.01), while 27.1% of patients in the drug group compared to 15% of the placebo group achieved clinical remission at week 8 (*p* = 0.10). In contrast, a statistically significant difference (*p* < 0.001) was observed when the composite outcome consisting of clinical response and a reduction of at least 50% in fecal calprotectin or CRP was taken into consideration (42.4% vs. 10%) [49].

Week 12 through week 112 was the open-label period in which all patients received 210 mg SC of brazikumab every 4 weeks. At weeks 8 and 24, 42.3% of patients who received MEDI2070 during both trial periods experienced a persistent clinical response compared to 23.1% of patients who received a placebo during the double-blind phase; prolonged clinical remission occurred in 23.1% and 11.5% of patients, respectively. Unfortunately, the study did not include endoscopic or instrumental outcomes [49].

At week 8 and 12 of the double-blind period, significantly greater reductions in fecal calprotectin (least squares mean difference of −105.6 and −124.6 with respective *p*-values of 0.027 and 0.034) and CRP concentrations (least squares mean difference of −17.6 and −10.8 with respective *p*-values of 0.007 and 0.008) were shown in patients treated with brazikumab compared with those receiving a placebo. Patients who continued taking the medication throughout the open-label period maintained these reductions, but those who took a placebo during the double-blind period experienced significant drops in fecal calprotectin (*p* = 0.001) and CRP concentrations (*p* = 0.002) from weeks 12 to 24 [49].

Another phase IIb/III study (INTREPID) and its respective open-label extension study were developed to evaluate brazikumab versus a placebo and an active comparator (adalimumab) for CD [50].

However, in June 2023, Astrazeneca announced that the brazikumab development program for the treatment of CD was discontinued. Astrazeneca’s decision was associated with the drug’s development timeline and was not secondary to safety concerns [51].

#### 4.2.2. Safety

At week 12, AEs occurred in 40 of 59 patients in the brazikumab group (67.8%) compared to 41 of 60 patients (68.3%) in the placebo group. SAEs were observed in 8% of patients in both the drug and placebo arms [49]. Clinically significant infections were reported in 4 patients in the drug arm vs. 11 patients in the placebo arm. In both research periods, 67.3% of patients receiving brazikumab experienced treatment-emergent AEs in the open-label phase (up to week 24) compared to 65.4% of patients receiving a placebo [49]. In the double-blind period, SAEs occurred in 7.7% of patients receiving a placebo and in 15.4% of patients receiving the drug during both study periods [49].

### 4.3. Guselkumab

#### 4.3.1. Efficacy

Guselkumab is an all-human monoclonal antibody (IgG1-lambda) already approved for the treatment of psoriatic arthritis [52,53].

The phase II trial GALAXI-1 investigated the potential of guselkumab as a treatment for patients with moderately to severely active CD who had either not responded well or were intolerant to conventional or biologic therapies [54]. The study aimed to assess both the effectiveness and safety of guselkumab in this patient population. In this trial the patients were randomized 1:1:1:1:1 to receive guselkumab at 200 mg, 600 mg, or 1200 mg IV; a placebo at weeks 0, 4, and 8; or ustekinumab at 6 mg/kg IV at week 0 and SC at 90 mg at week 8 (reference arm) [54]. The primary endpoint was a change from the baseline in the CDAI score at week 12 assessed by using the difference in the least squares means (LSMs). It was achieved in the entire guselkumab group (−160.4 in the 200 mg arm, −138.9 in the 600 mg arm, and −144.9 in the 1200 mg arm) compared with the placebo (−36.2) (*p* < 0.05 for all comparisons). Excellent results also were achieved at the secondary endpoints [54]. Clinical remission (CDAI < 150) at week 12 was observed in 54%, 58%, and 50% of patients, respectively, in the 200 mg, 600 mg, and 1200 mg groups vs. 16.5% of the placebo group (nominal *p* < 0.05), while a clinical response (a CDAI decrease of at least 100 points) was observed at week 12 in 70.5% (200 mg), 66.7% (600 mg), and 60.7% (1200 mg) compared to the placebo group (24.6% with nominal *p* < 0.05) [54]. Also, the rates of endoscopic response were significantly higher in patients treated with guselkumab (37.7%, 36.5%, and 32.8%) vs. the placebo (11.5%, nominal *p* < 0.05) [54].

In contrast, the study design did not include a direct comparison between the efficacy of ustekinumab and guselkumab, since ustekinumab was a reference arm. No long-term maintenance data are available [54].

In addition, GALAXI 2 and GALAXI 3 are two ongoing 48-week phase III confirmatory studies [55].

On the contrary, DUET-CD (NCT05242471) represents a phase II clinical trial designed to assess the effectiveness and safety of a combined treatment approach (specifically, the use of guselkumab and golimumab) in managing CD. However, no data are available yet [56].

#### 4.3.2. Safety

In the GALAXY1 trial, 60% of patients in the placebo arm, 43.8% in the 200 mg arm, 50.7% in the 600 mg arm, and 42.5% in the 1200 mg arm showed one or more AEs, while SAEs occurred in 5.7% (placebo group), 4.1% (200 mg group), 5.5% (600 mg group), and 1.4% (1200 mg group) [54]. The rates of infection were 21.4% in the placebo group and 12.3%, 17.8%, and 15.1% in the other groups. Finally, serum sickness or anaphylaxis were not experienced as severe hypersensitivity events. In addition, no opportunistic infections, deaths, or cases of active tuberculosis were documented through week 12 [54].

### 4.4. Mirikizumab

#### 4.4.1. Efficacy

Mirikizumab is another humanized IgG4 monoclonal antibody specific to the p19 subunit of IL-23.

Efficacy in CD was evaluated in a multicenter, randomized, double-blind, placebo (PBO)-controlled trial (SERENITY trial (NCT02891226)) [57]. In this trial, 191 patients with moderate-to-severe CD were randomized 2:1:1:2 to receive a placebo or 200 mg, 600 mg, or 1000 mg of mirikizumab IV every 4 weeks. Endoscopic response (defined as a 50% reduction from the baseline in the SES-CD score) at week 12 was the primary endpoint of the study [57]. The primary endpoint was achieved in every mirikizumab group compared with the placebo (10.9%): the 200 mg group (25.8%, *p* = 0.79), 600 mg group (37.5%, *p* = 0.03), and 1000 mg group (43.8, *p* < 0.001) [57]. Endoscopic remission (SES-CD score of <4 for ileal-colonic disease or <2 for isolated ileal disease and no subscore > 1) at week 12 was observed with statistically significant differences only in the 600 mg arm (15.6%, *p* = 0.032) and the 1000 mg arm (20.3%, *p* = 0.009) vs. placebo (1.6%) [57]. In the 200 mg arm, only 6.5% of patients (*p* = 0.241) showed endoscopic remission. For the clinical endpoint, such as CDAI response (decrease from the baseline in the CDAI score of 100 points or more or a CDAI score < 150) and CDAI remission (CDAI score of <150) at week 12, a significant improvement was observed in every group compared to the placebo in all clinical outcomes except for the 200 mg group in the CDAI remission (*p* = 0.406) [57]. All patients who achieved an improvement in the SES-CD score at week 12 were randomized 1:1 to receive mirikizumab IV (200 mg, 600 mg, or 1000 mg based on the dosage of drug received during the induction period) or mirikizumab at 300 mg SC every 4 weeks through week 52 (maintenance period) [57]. The endoscopic response rates at week 52 were 58.5% and 58.7% in the IV and SC groups, respectively, while endoscopic remission was achieved by 19.5% and 32.6%. Among the patients with endoscopic response at week 12, 50% (IV arm) and 64.3% (SC arm) of patients maintained endoscopic remission at week 52. The patients who did not achieve any improvement from the baseline of the SES-CD score at week 12 (both in the drug arm and placebo arm) started IV mirikizumab 1000 mg and placebo SC every 4 weeks through week 52 [57]. In these patients, the endoscopic response was 20.0% in patients who had not shown endoscopic improvement at week 12 and 42.4% in patients who received a placebo during induction and switched to mirikizumab (1000 mg) in maintenance. In contrast, the endoscopic remission rates at week 52 in the patients who had received mirikizumab and placebo in the induction part of the trial were 13.3% and 18.6%, respectively [57].

Two additional studies, the phase III VIVID 1 trial (NCT03926130) [58] and its open-label extension trial (VIVID 2 (NCT04232553)) [59] are ongoing. The first trial aims to assess both the effectiveness and safety of mirikizumab in individuals with CD when compared to both a placebo and an active comparator (ustekinumab) [58]. In the second trial, the goal is to assess the drug’s effectiveness and safety over an extended period in individuals with CD [59]. Anticipated data from the initial results are projected to be accessible by the year 2025.

#### 4.4.2. Safety

In the induction period, treatment-emergent adverse events (TEAEs) were reported in 58.1% in the 200 mg group and 65.6% in both the 600 mg and 100 mg groups compared to 70.3% of the placebo group; the most common AEs among the mirikizumab groups were headache (3.1%), worsening of CD (14.1%), arthralgia (4.7%), and nausea (3.1%) [57]. Regarding SAEs, three cases were reported in the 600 mg arm (chest pain, worsening of CD, colon perforation, and colonic stenosis), two 2 cases in the 1000 mg arm (abdominal and back pain), and no cases in the 200 mg arm. Instead, seven SAEs were listed in the placebo group (worsening of CD, pneumatosis intestinalis, malaise, hypokalemia, and pyrexia) [57].

Among the patients who achieved endoscopic improvement at week 12, during the maintenance period, no SAEs were reported in the patients in the mirikizumab IV group while two cases were reported in the patients that received mirikizumab (300 mg SC) after week 12 [57]. Instead, eight SAEs were reported at week 52 in the induction placebo group and three in the patients who received mirikizumab in the induction period but had not achieved endoscopic improvement at week 12. No cases of veno-occlusive illness (including pulmonary embolism), cancer, or deaths were recorded during the study’s induction or maintenance phases [57].

The efficacy and safety results for the main RCTs regarding IL-23-inhibitors are summarized in Table 1 and Table 2.

## 5. Discussion

Ustekinumab, an anti-IL-12/IL-23p40 agent, is the only medication in this class that is currently licensed for the treatment of CD and UC [60]. Among the IL-23p19 inhibitors, risankizumab showed a clinical and endoscopic response with a good safety profile and is currently the only drug approved by the FDA and EMA for CD [46] with an induction dose of 600 mg IV at week 0, 4, and 8 followed by 360 mg self-administered via SC injection with an on-body injector (OBI) at week 12 and every 8 weeks thereafter [44,61].

During phase II trials, mirikizumab, guselkumab, and brazikumab exhibited promising outcomes, displaying evident advantages over a placebo in both clinical response and (specifically for mirikizumab) an endoscopic response. However, development efforts for brazikumab in the context of CD were halted [51], while late-stage development programs are progressing for mirikizumab [57] and guselkumab [54], with expectations for their commercial availability in the near future.

Effectively placing these agents within treatment algorithms and making suitable choices regarding which patients should receive them will be of utmost importance.

In addition to efficacy, there are several reasons why IL-23 inhibitors are expected to acquire a central role in the treatment of CD.

For example, a strength of this drug category concerns the route of administration. Subcutaneous administration with no major concerns regarding injection site reaction increases adherence and long-term management of chronic diseases and is more tolerated than IV administration [62]. In this context, unlike anti-TNF agents, ustekinumab and risankizumab showed low rates of immunogenicity that may be related to maintenance of the response in CD and may lead to a reduced risk of infusion reactions [3,41,63].

Another strength concerns the safety profile. Although long-term data in CD are available only for a few drugs, experience with the use of IL-23 inhibitors in dermatology, such as risankizumab and guselkumab for plaque psoriasis and psoriatic arthritis, demonstrates an excellent long-term safety profile with no obvious increase in infectious or neoplastic or cardiovascular risks [64,65].

Finally, IL-23 inhibitors can be used in patients with extraintestinal manifestations (EIMs) of CD. Risankizumab [46] and guselkumab [66] are in fact currently approved by the EMA for the treatment of plaque psoriasis and psoriatic arthritis, which is why they may have a role in the management of patients with IBD and psoriasis [67].

Despite the advantages of IL-23 inhibitors, it is still unclear how this category of drugs can be positioned within the CD treatment algorithm. This decision can often be intricate, requiring collaborative decision-making approaches that take into account patient preferences, factors related to the disease’s characteristics, the presence of additional symptoms outside the digestive system, and the patient’s underlying health conditions [68].

TNF antagonists continue to hold a pivotal position in the management of CD, particularly in patients with high-risk disease phenotypes like those with fistulas, penetrating disease [69], or stricturing SB involvement [70]. Furthermore, the creation of biosimilars for anti-TNFs has increased the accessibility of this class of drugs by making them more cost-effective [71].

However, for patients with CD who do not experience a positive response to TNF inhibitors, IL-12/IL-23 inhibitors may present a promising alternative treatment option. Indeed, in this group of patients, there is a notable increase in the expression of genes associated with the IL-23R-dependent pathway when compared to responders [72]. These findings indicate that individuals who do not respond to TNF inhibitors could potentially benefit from targeted therapy focusing on IL-23 (ustekinumab or risankizumab) [72].

Moreover, the SEAVUE study revealed that both ustekinumab and adalimumab monotherapy demonstrated substantial rates of clinical remission in patients with CD, but ustekinumab did not achieve statistical significance in terms of superiority when compared to adalimumab [73]. This investigation offers valuable comparative data on the effectiveness and safety of two biological agents employing distinct mechanisms of action for treating CD. These results confirm that both mechanisms of action are suitable as first-line treatments for these patients [73].

Furthermore, risankizumab has demonstrated its superiority over ustekinumab in attaining clinical and endoscopic remission [48]. It is conceivable, therefore, that risankizumab could replace ustekinumab within the treatment algorithm in the future.

However, many issues are still open. For example, long-term efficacy and safety data of IL-23 inhibitors in CD are lacking. We also do not have data in special populations such as the elderly, children, pregnant women, and patients with perianal disease or undergoing surgery.

All available evidence comes from retrospective studies of these drugs in the treatment of extraintestinal diseases. In a retrospective study that enrolled patients aged ≥ 65 years with plaque psoriasis, risankizumab and guselkumab proved to be promising, safe, and effective options in elderly patients [74].

Few data are available regarding the use of these drugs in the pediatric population. Regarding CD, however, only a phase III trial is ongoing to evaluate the efficacy of mirikizumab in these patients (NCT04844606) [75].

As the array of novel biologics for treating patients with IBD continues to expand, the question of whether these new drugs with innovative mechanisms of action can be combined with conventional therapies remains without a definitive answer. Currently, no data are also available regarding dual therapies with selective IL-23 inhibitors. Only a study of a combination therapy with guselkumab and golimumab (a subcutaneous TNF-α inhibitor) in patients with CD is ongoing [76]. It is a plausible consideration that utilizing IL-23 inhibitors in combination therapy could be a favorable option, particularly due to their favorable safety profile.

## 6. Conclusions

The proven effectiveness of ustekinumab in blocking IL-12/IL-23 serves as a promising foundation for the development of agents specifically targeting IL-23. Currently, only risankizumab has obtained approval for the treatment of CD, while the second generation of selective anti-IL-23 therapies showed a good efficacy profile. Additionally, consistent with data from various indications, the safety profiles of all anti-IL-23 agents seem notably favorable. There is no apparent elevation in the risk of infections, and no significant signals related to neoplastic risks or cardiovascular complications were identified across studies of IBD. The subcutaneous maintenance administration of IL-23-selective monoclonal antibodies is generally well-tolerated, with no major concerns regarding injection site reactions. However, the consistent applicability of these agents across all patient groups will depend on the outcomes of ongoing controlled research studies. Precision medicine research seeks to offer a more profound comprehension of the mechanisms by which these substances operate and how they can potentially enhance or collaborate with other biological or small-molecule treatments. This knowledge will enhance the precise placement of these therapies and help guide personalized therapeutic decisions based on individual patient backgrounds.

## Figures and Tables

**Table 1:** Summary of efficacy of IL-23 inhibitors in Crohn’s disease. IV: intravenous; SC: subcutaneous; CDAI: Crohn’s Disease Activity Index; SES-CD: Simple Endoscopic Score for Crohn´s Disease.

Drug (Study, Phase)	Patients	Route of Administration	Dosing	Frequency of Dosing	Primary Endpoint	Primary Endpoint Results
Guselkumab (GALAXI-I, phase II)	73 (200 mg) 73 (600 mg) 73 (1200 mg) 71 (ustekinumab) 70 (placebo)	IV induction, SC at W8	Guselkumab (200 mg, 600 mg, or 1200 mg); ustekinumab 6 mg/kg at W0 and 90 mg SC at W8; placebo	Guselkumab: q4W Ustekinumab: q8W	Change from baseline of CDAI score at W12	- 160 (200 mg) - 139 (600 mg) - 145 (1200 mg) - 136 (ustekinumab) - 36 (placebo)
Brazikumab (phase II)	59 (700 mg) 60 (placebo)	IV W0 and W4, SC every 4Ws starting at W12	700 mg brazikumab; placebo	700 mg IV q4W; 210 mg SC starting at W8	Clinical response (a CDAI decrease of 100 points from baseline) at W8; clinical remission (CDAI < 150) at W8	Clinical response: 49% (brazikumab) 27% (placebo) Clinical remission: 27% (brazikumab) 15% (placebo)
Mirikizumab (phase II)	31 (200 mg) 35 (600 mg) 64 (1000 mg) 64 (placebo)	IV	Mirikizumab (200 mg, 600 mg, 1000 mg); placebo	q4W	Endoscopic response (a 50% reduction from baseline in SES-CD score) at W12	26% (200 mg) 37% (600 mg) 44% (1000 mg) 11% (placebo)
Risankizumab (ADVANCE, phase III)	Conventional/biologic therapy failure: 336 (600 mg) 339 (1200 mg) 175 (placebo)	IV	Risankizumab (600 mg, 1200 mg); placebo	q4W	Clinical remission (CDAI < 150 in US/based on patient-reported stool frequency and abdominal pain in non-US countries) at W12 Endoscopic response (>50% decrease in SES-CD from baseline) at W12	Clinical remission 45%/43% (600 mg) 42%/41% (1200 mg) 25%/22% (placebo) Endoscopic response 40% (600 mg) 32% (1200 mg) 12% (placebo)
Risankizumab (MOTIVATE, phase III)	Only biologic therapy failure: 191 (600 mg) 191 (1200 mg) 187 (placebo)	IV	Risankizumab (600 mg, 1200 mg); placebo	q4W	Clinical remission (CDAI < 150 in US/based on patient-reported stool frequency and abdominal pain in non-US countries) at W12 Endoscopic response (>50% decrease in SES-CD from baseline) at W12	Clinical remission: 42%/35% (600 mg) 40%/40% (1200 mg) 20%/19% (placebo) Endoscopic response: 29% (600 mg) 34% (1200 mg) 11% (placebo)
Risankizumab (FORTIFY, phase III)	Patients who responded to the 12W induction treatment in either ADVANCE or MOTIVATE: 141 (360 mg) 157 (180 mg) 164 (placebo)	SC	Risankizumab (360 mg, 180 mg); placebo	q8W	Clinical remission (CDAI < 150) at W52; endoscopic response (50% decrease in SES-CD from baseline) at W52	Clinical remission: 52% (360 mg) 55% (180 mg) 41% (placebo) Endoscopic response: 47% (360 mg) 47% (180 mg) 22% (placebo)

**Table 2:** Summary of safety of IL-23 inhibitors in Crohn’s Disease. AE: adverse event. SAE: serious adverse event.

Drug (Study, Phase)	Patients	Total AEs: Patients or Events	Total SAEs: Patients or Events	Infections: Patients or Events	Serious Infections: Patients or Events
Guselkumab (GALAXI-I, phase II)		At W12: patients	At W12: patients	At W12: patients	At W12: patients
	73 (200 mg)	32 (43.8%)	3 (4.1%)	9 (12.3%)	1 (1.4%)
	73 (600 mg)	37 (50.7%)	4 (5.5%)	13 (17.8%)	2 (2.7%)
	73 (1200 mg)	31 (42.5%)	1 (1.4%)	11 (15.1%)	0 (0%)
	71 (ustekinumab)	36 (50.7%)	4 (5.6%)	9 (12.7%)	1 (1.4%)
	70 (placebo)	42 (60%)	4 (5.7%)	15 (21.4%)	0 (0%)
Brazikumab (phase II)		At W12: patients	At W12: patients	At W12 (no distinction regarding severity): patients	NA
	59 (700 mg)	40 (67.8%)	5 (8.5%)	4 (6.7%)	
	60 (placebo)	41 (68.3%)	6 (10%)	7 (11.6%)	
Mirikizumab (phase II)		At W12: patients	At W12: patients	NA	NA
	31 (200 mg)	18 (58.1%)	0 (0%)		
	35 (600 mg)	21 (65.6%)	3 (9.4%)		
	64 (1000 mg)	42 (65.6%)	0 (0%)		
	64 (placebo)	45 (70.3%)	7 (10.9%)		
Risankizumab (ADVANCE, phase III)	Conventional/biologic therapy failure:	At W12: events (rate)	At W12: events (rate)	NA	At W12: events (rate)
	336 (600 mg)	210 (56%)	27 (7%)		3 (1%)
	339 (1200 mg)	191 (51%)	14 (4%)		2 (1%)
	175 (placebo)	105 (56%)	28 (15%)		7 (4%)
Risankizumab (MOTIVATE, phase III)	Only biologic therapy failure:	At W12: events (rate)	At W12: events (rate)	NA	At W12: events (rate)
	191 (600 mg)	98 (48%)	10 (5%)		1 (<1%)
	191 (1200 mg)	121 (59%)	9 (4%)		2 (1%)
	187 (placebo)	137 (66%)	23 (13%)		5 (2%)
Risankizumab (FORTIFY, phase III)	Patients (at least one dose) who responded to the 12W induction treatment in either ADVANCE or MOTIVATE:	Patients:	Patients:	NA	Patients:
	179 (360 mg)	129 (72%)	24 (13%)		8 (4%)
	179 (180 mg)	128 (72%)	22 (12%)		5 (3%)
	184 (placebo)	135 (73%)	135 (73%)		7 (4%)

## Data Availability

No new data were generated or analyzed in support of this research.
